# Impact of Gingivitis on Circulating Neutrophil Reactivity and Gingival Crevicular Fluid Inflammatory Proteins

**DOI:** 10.3390/ijerph19106339

**Published:** 2022-05-23

**Authors:** Helen M. Roberts, Zehra Yonel, Alpdogan Kantarci, Melissa M. Grant, Iain L. C. Chapple

**Affiliations:** 1 School of Dentistry, Institute of Clinical Science, University of Birmingham and Birmingham Community Healthcare Foundation Trust, 5 Mill Pool Way, Edgbaston, Birmingham B5 7ET, UK; helenroberts1988@gmail.com (H.M.R.); z.yonel@bham.ac.uk (Z.Y.); i.l.c.chapple@bham.ac.uk (I.L.C.C.); 2 Forsyth Institute, 245 First Street, Cambridge, MA 02142, USA; akantarci@forsyth.org

**Keywords:** gingivitis, neutrophil, inflammation, gingival crevicular fluid, reactive oxygen species, neutrophil extracellular traps, cytokines, chemokines, chemotaxis

## Abstract

Gingivitis is an extremely common oral inflammatory condition and can be induced in humans using an acute 21-day experimental gingivitis model. Neutrophils are known to be highly prevalent in the gingival crevice during gingival inflammation; however, the effect of gingivitis and the associated biofilm on peripheral blood neutrophils (PBN) is not well characterised. Thus, the aim of this study was to examine the impact of inflammation induced by experimental gingivitis and its resolution upon the function of PBN. Fifteen systemically healthy volunteers undertook a split-mouth 21-day experimental gingivitis study followed by a resolution phase of 14 days. PBN function, including reactive oxygen species (ROS) production, neutrophil extracellular trap (NET) release, directional chemotactic accuracy and expression of host mediators in gingival crevicular fluid (GCF), were measured at baseline (day 0), on day 21 and on day 35. NET formation and ROS production were significantly elevated at day 21. Chemotactic speed was also elevated in response to bacterial peptide fMLP at day 21. At day 35, ROS production in response to an Fcgamma stimulant, opsonised *Staphylococcus aureus*, remained elevated. The data presented suggest a lasting biological impact of the experimental gingivitis on PBN function even after clinical symptoms have abated.

## 1. Introduction

The periodontal tissues lie adjacent to the teeth and are crucial structures for maintenance of the dentition. The teeth, having a non-shedding surface, continuously accumulate a dental plaque biofilm, and if not removed daily through tooth brushing, the biofilm induces inflammation of the adjacent gingival tissues, termed plaque-induced gingivitis [1]. It is characterised by localised redness and swelling, but the absence of changes such as spontaneous bleeding or periodontal attachment loss [1]. Removal in or reduction of the plaque stimulus allows for an apparent return of inflammation back to a healthy state, as judged clinically, and thus, gingivitis is classified as a reversible condition. Gingivitis is highly prevalent, with estimates between 32–56% in adults [2,3], though these were hampered until recently due to the lack of a true case definition [1], which is of importance because chronic inflammation of the gingiva is a necessary prerequisite for periodontitis in susceptible people [4].

The measurement of gingival or periodontal inflammation is traditionally made through clinical observation of the gingiva. Indices to estimate the macroscopic changes occurring in inflammation, such as redness and swelling, or the mechanical stimulation of the tissues to induce bleeding were introduced nearly five decades ago [5] and record symptoms of gingivitis at a clinical level without regard for underlying biological processes. Thus, they may be viewed as crude and insensitive to dynamic processes occurring in the tissues. It was recently recognised that “pristine health” does not exist as classically described in textbooks, with clinical health accommodating a degree of immune surveillance and mild localised clinical signs of inflammation [6].

The inflammatory response is dominated by the neutrophil as the most abundant leukocyte in circulation, and also in the gingival tissues in response to plaque accumulation [7]. Neutrophils enter the tissues from the blood stream in response to chemotactic agents of both endogenous (e.g., interleukin-8/CXCL8) and exogenous (e.g., N-formyl-methionyl-leucyl-phenylalanine; fMLP) origin, which accumulate in the inflamed periodontal tissues. Neutrophils possess a powerful antimicrobial repertoire, including the generation of reactive oxygen species (ROS), which can occur during phagocytosis and frustrated phagocytosis [8], neutrophil extracellular trap (NET) production, a terminal defence mechanism involving the extrusion of DNA in association with antimicrobial peptides that trap bacteria in tissues [9], and the release of vesicular antimicrobial peptides held within the cytoplasmic granules [10]. Neutrophil killing activity can be enhanced by priming, a process that involves pre-exposure of the cell to an agent that enhances the response of cells to a secondary stimulating agent, such as bacteria and their products. Priming occurs during cell transit from circulation to the site of inflammation, readying the cell for maximal killing behaviour. From the gingival tissue neutrophils can enter the gingival crevice and thence the gingival crevicular fluid, where they can make up 90% of the cell population [7].

Inflammation can alter not only neutrophils at the site of inflammation but also in the circulation; previously, peripheral blood neutrophils (PBNs) from patients with periodontitis have been shown to be both hyperactive and hyper reactive [11,12] in terms of ROS generation [11,12,13], pro-inflammatory cytokine release [14], impaired chemotaxis [15] and impaired NET production [16]. However, it is unknown whether gingivitis elicits a change in PBN phenotype or how long such a change takes to resolve, both of which may be relevant to the specific risk for periodontitis that some patients appear to exhibit. The use of a human model of gingivitis, such as a modified version of the 21-day experimental gingivitis model, could shed light on these questions: animal models may not be fully relevant due to the complex interactions between the host immune response and the microbiome. 

Thus, it was hypothesised that PBN may serve as surrogate markers of subclinical gingival inflammation and its resolution. Therefore, in this pilot study the functional characteristics of PBN were examined alongside the inflammatory load, as measured by cytokines, as biological outcomes of experimental gingivitis, using a modified model of experimental gingivitis in humans.

## 2. Materials and Methods

### 2.1. Study Population

The experimental gingivitis model employed was previously described by Chapple et al. [17] and the study was approved by the South Birmingham Local Research Ethical Committee (LREC 2004/074) and all volunteers provided written informed consent. Fifteen never smoking volunteers (9 females, 6 males, mean age ± SD = 22 ± 3 years) with unremarkable medical histories, no periodontitis (past or present) and not undergoing orthodontic or prosthetic appliance therapy or taking antimicrobial or anti-inflammatory medications were enrolled in the 35-day study. 

A soft vinyl mouth guard was fabricated to cover the maxillary 24, 25 and 26 teeth (all volunteers were right-handed) to be worn during brushing, in order to shield the area from mechanical or chemical plaque removal. The maxillary 14, 15 and 16 teeth were observed for comparison to ensure accumulation of plaque at the test teeth (undergoing normal hygiene practices, alongside the rest of the mouth) and termed control teeth. For the entirety of the study, volunteers were asked to refrain from chewing gum or using mouthwashes. The study period consisted of an initial pre-baseline assessment (day-14), at which time alginate impressions were taken for the tooth-shield construction and the teeth were professionally scaled in order to achieve close to pristine gingival health by the baseline (day 0) appointment. At day-14 the participants were also given oral hygiene instruction; no restrictions on choice of toothbrush or toothpaste were made. At baseline, clinical measures were recorded alongside GCF sampling and venipuncture. The tooth-shield was then worn during brushing for the experimental gingivitis phase of 3 weeks (up to day 21), after which plaque was removed by prophylaxis and normal oral hygiene practices resumed. Participants were assessed again following resolution of the experimentally induced gingival inflammation phase two weeks later (day 35). Gingival inflammation and plaque accumulation were assessed throughout the study on the test and control teeth using the gingival index (GI) of Löe et al. [18] and the modified Quigley–Hein Plaque Index (PI) [19], respectively. Data are expressed as percentage of the data above: 0 for gingival index and 1 for plaque index, as these points represented clinically healthy scores. All volunteers enrolled in the study were treated on the same days between 9–11 am to ensure study consistency (one dentist performed all the examinations ILC) and reduce experimental bias.

In addition, a cohort of 10 systemically healthy never smoking volunteers not undergoing experimental gingivitis were enrolled under the same selection criteria and donated blood samples for PBN isolation, which were used in chemotaxis assays using GCF as the chemoattractant.

### 2.2. Gingival Crevicular Fluid (GCF) Collection and Processing

Gingival crevicular fluid (GCF) was collected on Periopaper strips (Oraflow, Plainview, NY, USA) placed in the crevice of each tooth (24, 25, 26 and 14, 15, 16) at the mesio-buccal aspect for 30 s as previously described [20]. GCF volumes were measured using a pre-calibrated Periotron 8000 (Oraflow, Plainview, NY, USA). The three test strips (one from each of the test/control teeth in each participant) were pooled in PBS-BSA (0.05%, 200 μL) and incubated at room temperature for 30 min to allow the GCF to elute, after which the strips were trapped within the lid of each tube. After centrifugation (10 min, 2000 rcf; SciSpin micro, Geneflow, UK), the lid and strips were removed and the GCF diluent was filter sterilised using 0.22 μm syringe filters (Gilson Scientific, Luton, UK, ANV1322) to remove any cell debris/microbes and stored at −80 °C.

### 2.3. Collection of Blood and Preparation of Neutrophils

For each blood donation, approximately 24 mL of venous blood was collected from the ante-cubital fossa into VacutainerTM lithium heparin (17 IU/mL) tubes, and PBNs were isolated using Percoll density gradients (GE Healthcare, Chicago, IL, USA) as previously described [11]. Cell viability, typically >98%, was determined by dye exclusion (trypan blue). Cell purity was determined by cytospin.

### 2.4. Enhanced Chemiluminescent Assay

Chemiluminescence assays were performed on isolated PBNs at day 0, 21 and 35 using luminol to detect total ROS as previously described [15]. GM-CSF was used where described at 10 ng/mL 30 min prior to stimulation with *Fusobacterium nucleatum* (ATCC 10953; 6.8 × 109 bacteria/mL) at a multiplicity of infection (MOI) of 300:1, Opsonised *Staphyloccocus aureus* (NCTC 6571; 1.69 × 109 bacteria/mL) MOI of 300:1, or phorbol myristate acetate (PMA; 25 mM) at 37 °C until the peak ROS output was recorded. 

### 2.5. Neutrophil Extracellular Trap (NET) Quantification Assay

NET quantification was performed as previously described [21]. Cells were stimulated with unopsonised *S. aureus* (MOI of 150:1), PMA (50 nM) or HOCl (0.75 mM) at 37 °C for 3 h. 

### 2.6. Chemotaxis Assays

Chemotaxis was visualised over 20 min at room temperature using the Insall Chamber as previously described [15,22]. Chemoattractant fMLP (10 nM) or CXCL8 (200 ng/mL), GCF sample (standardised to 25 ug/mL after protein content was determined by bicinchoninic acid assay [23] by dilution in PBS) or control (PBS) were used.

### 2.7. GCF Cytokine/Enzyme Quantification by Multiplex Immunoassay

The protein content of GCF samples was measured using the bicinchoninic acid (BCA) protein assay as previously described [23] and diluted in PBS to normalise the samples to 25 ug/mL. Host immune mediators including cytokines, chemokine and enzymes (IL-1β, IL-6, CXCL8/IL-8, MIP-1α, TNF-α, C5/C5a, C3a, CXCL1/Gro-a, CXCL2/Gro-b, CXCL5/ENA-78, MPO, S100A8 and S100A9) were measured in the GCF using bead-based multiplex assays (EMD Millipore, Burlington, MA, USA and R&D Systems, Minneapolis, MN, USA) and the Bioplex-200 Analyzer (Bio-Rad Laboratories, Hercules, CA, USA) according to the manufacturer’s instructions.

### 2.8. Statistical Analysis

All data were analysed using GraphPad Prism (version 5.0; GraphPad Software, San Diego, CA, USA). Normal distribution of data was determined using the Kolmogorov–Smirnov test. For GCF collection and processing a paired T-test was performed. GI/PI measurements, NETs, ROS and chemotaxis assays were analysed using a one-way ANOVA and Tukey’s post-test. Chemotaxis assays using GCF was analysed by a Wilcoxon test. Heatmaps was created from the multiplex results of the GCF samples were analysed using ClustVis [24]: data were ln(x + 1) transformed rows and columns were clustered using correlation distance and average linkage.

## 3. Results

To ensure that gingivitis was induced clinically at three teeth over the 21 days of induction and then resolved clinically over 14 days, test and control sites were clinically monitored in each participant. Table 1 demonstrates that there were significant increases in clinical signs of inflammation at test sites measured by plaque index, gingival index and GCF volume at day 21 but not at day 35, when compared to contralateral control sites. As both test and control sites returned to clinical baseline values this suggests the clinical efficacy of the oral prophylaxis and resumption of self-performed oral hygiene at day 21 as an intervention for gingivitis [14].

**Figure 1:**
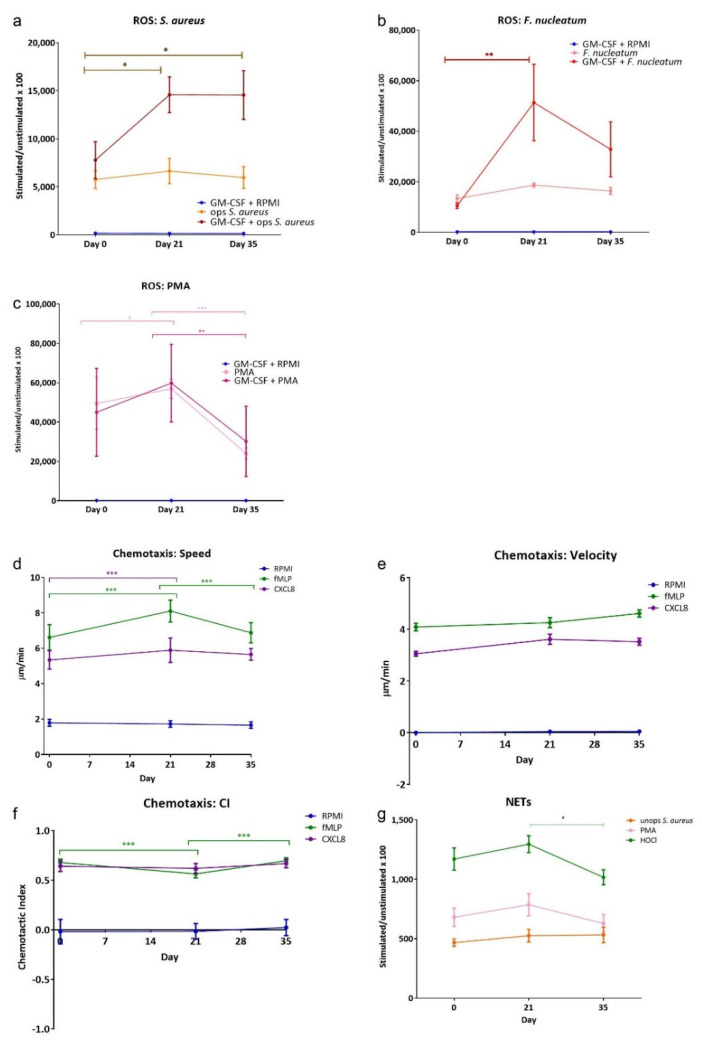
ROS, chemotaxis and NETs from gingivitis study volunteers. ROS production, chemotaxis and NET quantification in response to stimuli. Peak values for total ROS stimuli included: (**a**) opsonised *S. aureus* (MOI 1:300; Fcγ receptor stimulant); (**b**) *F. nucleatum* (MOI 300:1; TLR stimulant), (**c**) PMA (25 mM). Chemotaxis analysis was used to generate average (**d**) speed, (**e**) velocity and (**f**) chemotactic index (CI) for neutrophils from gingivitis study volunteers in the presence of RPMI (negative control), fMLP (10 nM) and CXCL8 (200 ng/mL) chemoattractants. NET production (**g**) was measured following stimulation with unopsonised *S. aureus* (MOI 150:1), PMA (50 nM) and HOCl (0.75 mM; direct stimulant). Average Relative Light Unit (RLU) and Arbitrary Fluorescent Unit (AFU) readings were normalised by dividing all values by the respective RPMI control for a given time point × 100. Data are presented as mean, min and max values ± standard error of the mean (SEM). Statistical test: ANOVA with Tukey’s post-test. * *p* < 0.05, ** *p* < 0.01 and *** *p* < 0.001.

Figure 1 shows the results from the three neutrophil functional assays employed. Total ROS release (Figure 1a–c) from PBNs isolated from participants with induced gingivitis was measured in the presence and in the absence of the priming agent GM-CSF (10 ng/mL). Priming elicited a greater release of ROS with all stimuli (i.e., PMA, *F. nucleatum* and opsonised *S. aureus*, as expected, but not priming agent GM-CSF on its own. There were increases in ROS production at day 21, and this remained elevated with both of these physiological stimuli at day 35. These data suggest a change in circulating neutrophil ROS production can be induced when as few as three teeth accumulate plaque and develop gingival inflammation.

Three parameters of chemotaxis of isolated PBNs from each participant were measured at each time point (Figure 1d–f). Speed and velocity of movement towards the bacterial peptide fMLP was greater than towards CXCL8, as expected. PBN speed (movement in any direction) was found to be significantly increased at day 21 for both chemoattractants, compared to day 0. This returned to day 0 levels after resolution of inflammation, measured at day 35. This pattern was not apparent for velocity (movement towards the chemoattractant); however, the chemotactic index (a measure of directional accuracy) demonstrated small but significant decrease at day 21 for fMLP. These results imply localised gingivitis-induced negative changes in PBN directional movement, which was normalised following resolution of inflammation. Quantification of NET production (Figure 1g) showed significant differences between day 21 and day 35 with HOCl (a direct stimulant of NET production that is non-receptor mediated), indicating that NET production was higher at day 21; a similar trend was seen with PMA.

**Figure 2:**
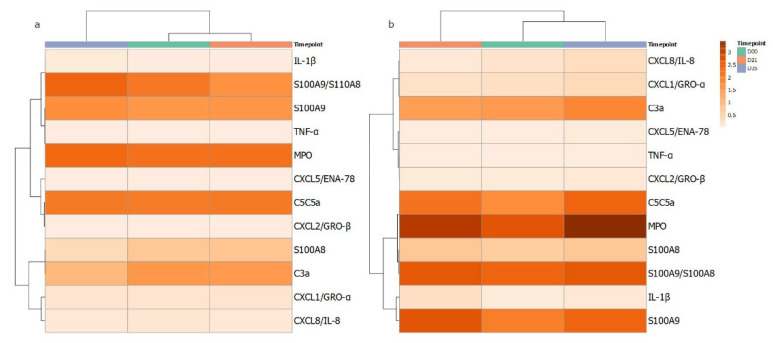
Heatmap of cytokines quantified from GCF samples in gingivitis. Measured mediators were grouped according to correlations in changes over the course of the 35 days. The darker the orange colour reflects higher level of the corresponding measured host mediator for (**a**) control and (**b**) test GCF collection sites at day 0, 21 and 35.

In parallel to the assessment of circulating neutrophil function, the local production of cytokines and chemokines was measured in GCF isolated from the gingival crevice of the test and control teeth. The data for the eleven analytes are shown in Table 2 and Supplementary Figure S1. For IL-1β and S100A9, significant increases were seen at day 21. However, for many analytes, significant increases were seen at day 35, following clinical resolution: C3, C5, CXCL1, CXCL5, CXCL8 and TNFα. An apparent pattern of the cytokine profiles over the gingivitis time course from GCF was investigated further by correlation-based clustering of the profiles’ mean data (Figure 2) of control and test sites. The different patterns visualised suggest that the cytokine/enzyme mediator profiles are associated with levels of inflammation as the patterns are different for the test and control sites. It could be speculated that the clustering of MPO, S100A8, S100A9, IL1b and S100A8/A9 suggests more neutrophils degranulating in the gingival crevice or tissue adjacent contributing to the content of the GCF.

**Figure 3:**
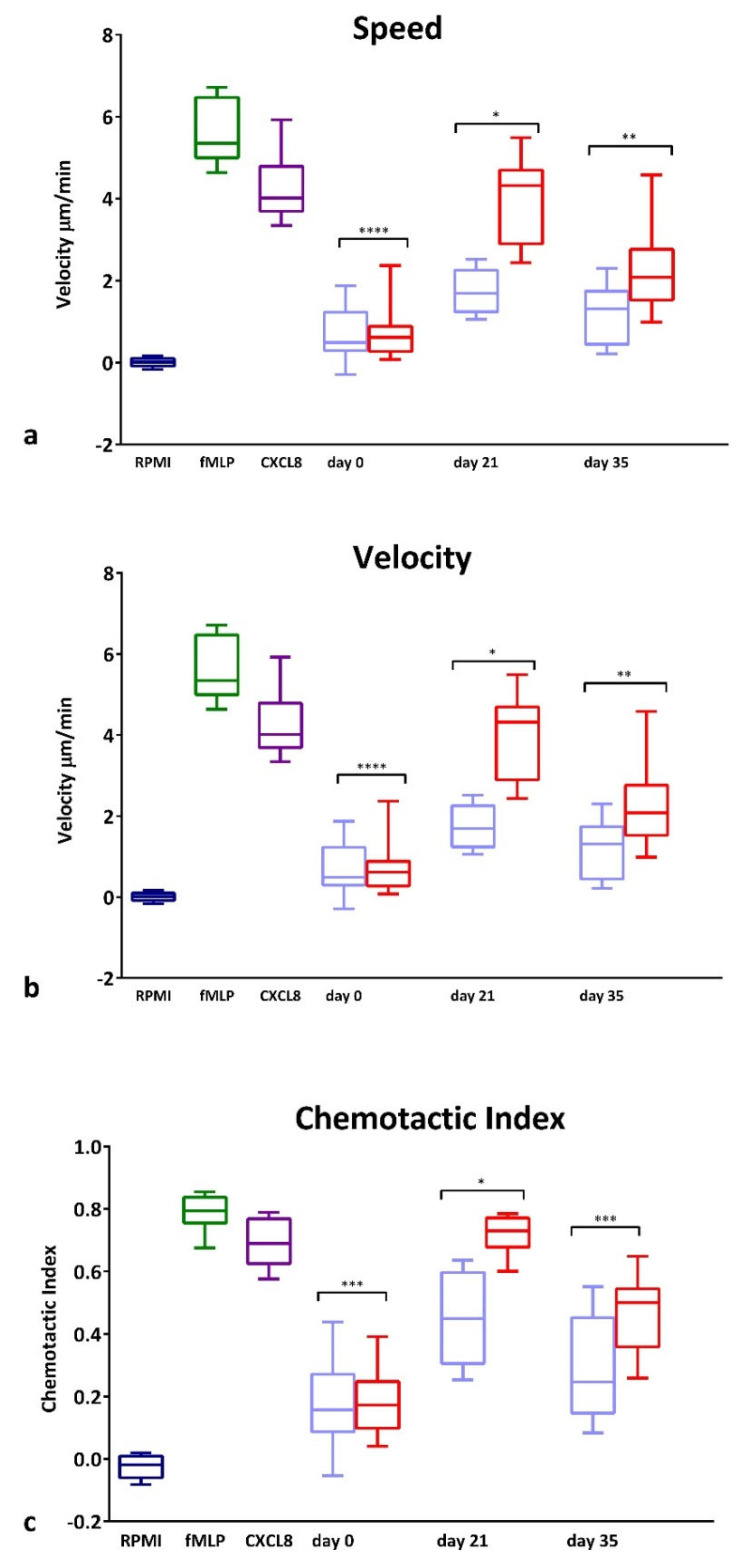
PBN Chemotaxis towards GCF from experimental gingivitis volunteers. Chemotaxis of PBNs isolated from periodontally and systemically healthy blood donors. Chemotaxis analysis was used to generate average (**a**) speed, (**b**) velocity and (**c**) chemotactic index (CI) for neutrophils in the presence of RPMI (negative control, dark blue), fMLP (10 nM, dark green) and CXCL8 (200 ng/mL, dark purple) chemoattractants or towards GCF diluted to 25 ug/mL. Data presented in light blue (at day 0, 21 and 35) show GCF collected from control side and data presented in red show GCF collected from test side used as chemoattractant. Statistical test: Wilcoxon test to compare test and control GCF locations. * *p* < 0.05, ** *p* < 0.01, *** *p*<0.001, **** *p* < 0.0001.

GCF collected from the test and control sites of the experimental gingivitis volunteers was used as a chemoattractant for PBNs isolated from 10 additional periodontally and systemically healthy blood donors. GCF was diluted to contain the same amount of protein (25 μg/mL) such that the different composition rather than the overall cytokine quantity could be assessed. Figure 3 illustrates speed (Figure 3a), velocity (Figure 3b) and CI (Figure 3c) of the PBNs exposed to GCF samples taken at day 0, 21 and 35. There were no differences at day 0 between the test and control site GCF samples; however, at day 21, GCF from test sites caused a significant increase in all three parameters (*p* < 0.0001) compared with corresponding control sites. At day 35, a significant difference was still evident for velocity (Figure 3b; *p* < 0.05) and CI (*p* < 0.01) between control and test sites demonstrating the effectiveness of collected GCF from inflamed sites, to induce chemotaxis.

## 4. Discussion

Alterations in peripheral blood neutrophil (PBN) function have previously been reported in periodontitis patients [11,12,13,14,15,16,25]. Gingivitis is a precursor to periodontitis and extremely prevalent in humans globally in susceptible patients, yet specific biological mechanisms have not been determined. We hypothesised that 21 days of plaque accumulation, creating mild localised gingivitis, would change the functionality of peripheral neutrophils and that the inflammatory signature would persist beyond clinical resolution of inflammation. The use of the split mouth model in otherwise healthy volunteers was employed to ensure consistency between volunteers, controlling the timeline of inflammation progression and resolution to enable intra-individual comparisons to be made between healthy and inflamed gingiva sites.

The induction of 21-day gingivitis was evident according to increases in gingival and plaque indices in addition to increased GCF volume and protein content at test sites relative to the healthy control sites. These data are consistent with previous studies, which demonstrate GCF volumes associate with disease severity [26,27,28], and may represent a more reliable marker of disease than other clinical markers of gingival inflammation, such as bleeding on probing and probing pocket depths [29]. By day 35, inflammation was resolved according to these oral hygiene measurements; however, this was not demonstrated for all assays measured in this study

NET formation measured as the release of nuclear DNA from PBNs in the gingivitis study volunteers showed all stimuli increasing NET production at day 21, with significance for HOCl, which then normalised by day 35. Neutrophils and NET-associated proteins have previously been identified within the supragingival plaque of individuals partaking in the experimental gingivitis model, demonstrating that biofilms are composed of both microbial and host immune cells and their products [30]. The results presented here demonstrated a reversible systemic effect of mild localised gingivitis on PBNs; a striking observation given the gingivitis was only induced at three maxillary teeth.

ROS production from isolated PBNs over the course of the 21-day gingivitis study showed an increase at the height of inflammation (day 21) and in the presence of the priming agent GM-CSF with PMA stimulus, although increases from day 0 to day 21 were visible, but not significant, with the other stimuli employed. These results demonstrate the potential for localised acute inflammation to enhance neutrophil responsiveness to stimuli such as bacteria and their products, which have varied receptor binding capabilities (e.g., TLR for *F. nucleatum* and FcγR for opsonised *S. aureus*), and to affect a stronger response following priming. The reinstatement of hygiene practices and consequent clinical resolution of inflammation caused a reversal in the elevated ROS production. ROS generation in periodontitis patients prior to and following clinical treatment has been previously reported [13], with superoxide release significantly higher in patient’s pre-treatment than controls with such differences lost following treatment when compared with healthy matched controls.

The chemotaxis data of PBNs from gingivitis study participants demonstrated greater overall cell speed at day 21, but lower CI compared with baseline and resolution using the chemoattractant fMLP. Neutrophil chemotaxis in periodontitis patients has been previously reported, with pre-treatment PBNs demonstrating reduced speed, velocity and CI than healthy volunteer PBNs for the same chemoattractants (fMLP and CXCL8) employed here [15].

GCF carries proteins from the gingival tissues and fluid via the crevice, which may provide insights into the immunological processes occurring within the gingival tissues [31,32]. Numerous studies have reported raised levels of immune mediators in experimental gingivitis for GCF, including transforming growth factor beta (TGFβ) [32] and IL-1α [33]. Previous work detailing proteomic analysis of GCF detected temporal changes in numerous protein levels during the course of experimental gingivitis, such as neutrophil defensins, which were elevated at Day 35 in test sites [34]. Host immune proteins, other mediators and cytokines were detected in the collected GCF using a multiplex assay. Increased concentrations of pro-inflammatory mediators were detected in test site samples over control sites; however, for some, this is shown at day 35 test instead of the expected day 21, and which was significant for C5/C5a, C3a, CXCL1/GRO-α, CXCL5/ENA-78, TNF-α and CXCL8. A non-significant increase was seen in MPO at day 35, as previously reported by Nascimento et al. [35] in saliva from participants of experimental gingivitis. These mediators are known neutrophil chemoattractants. This indicates that even though clinically the tissues appeared to have resolved inflammation, there is still a subclinical inflammatory presence that may be attracting PBNs into the area. Of interest is the finding of Wellappuli et al. [36], who explored the quantity of oral neutrophils, i.e., those found in saliva, over the course of a 21-day experimental gingivitis experiment with 2 weeks of resolution: they found that the number of oral neutrophils also remained significantly elevated at day 35. These authors suggest that there may be a persistent change in the capillary beds, allowing for the continued presence of neutrophils in the oral cavity following clinical resolution of inflammation; however, this needs further investigation. GCF flows directly into saliva and is the likely source of the neutrophils. However, there is a great dilution due to the change in volume. Furthermore, when measuring the chemotactic potential of the collected GCF against PBNs from a cohort of healthy individuals, strong chemotactic responses continued at day 35. Further research is required to understand if these chemoattractive molecules are active in situ and if there is also a sustained increase in the numbers of neutrophils in the gingival tissues at day 35.

The results presented here point towards a potential inflammatory profile whereby certain mediators are increased but resolve (e.g., IL-1β), whereas others do not (e.g., TNF-α). One hypothesis to explain these data is that individuals whose inflammatory profile reflects the latter group, who do not respond to gingivitis treatment may go on to develop periodontitis. Further analysis into the potential for mediator profiling to predict disease progression is an interesting potential research avenue. The results also demonstrate how biological elements of the resolution of inflammation and inflammatory responses can persist beyond clinical resolution of inflammation. Indeed, the role of the host in the potential for a prolonged inflammatory response after the removal of the main bacterial challenge should not be overlooked. There is the possibility that lifestyle and dietary choices as well as epigenetic changes may influence this phenotype [37].

The presence of acute systemic infections has been shown to affect the incidence and progression of chronic inflammatory disease; gingivitis causes a local, transient clinical inflammatory episode that may result in sufficient bacteraemia during physiological functions to generate an acute-phase response [38]. Gingivitis, if left unresolved, gives rise to a state of low-level chronic inflammation progression and the potential for progression to periodontitis.

It should be noted that the experimental gingivitis model employed in this study is a modified version of the original by Loë et al. [18]: only three teeth were shielded during brushing. The quantity of biofilm accumulation and associated inflammation may be similar to that of a few sites persistently missed in a mouth rather than a total lack of brushing; even so, it was apparent the gingivitis was induced at these sites that this quantity of inflammation caused changes to PBN functions. This may reflect the impact of transient bacteraemia upon PBN function, or local neutrophils becoming primed by local inflammatory mediators, prior to returning to the circulation and disseminating systemically.

Limitations of this study include a low participant number, a short inflammatory episode relative to the incidence of gingivitis in patient populations and a short follow-up period (although this has been deemed as the clinically acceptable end point according to the experimental model). Further studies that measure cytokines beyond day 35 of the gingivitis model would provide more information regarding the temporal relationship between the clinical and the biological resolution of inflammation and to explore the possibility of cohorts with distinct inflammatory profiles that may exist within a clinical diagnosis of gingivitis. In addition, cluster analysis of such measured mediators in greater numbers of volunteers would provide greater insight into a potential profile for inflamed versus non-inflamed conditions; moreover, measurement of circulating (i.e., serum) mediators may reveal additional insights when correlated to neutrophil behaviour.

## 5. Conclusions

In conclusion, this study has demonstrated that 21 days of plaque accumulation in systemically healthy volunteers induces mild localised gingivitis, which was associated with alterations in ROS, NETs and chemotaxis in peripheral blood neutrophils and additional changes in pro-inflammatory mediators in gingival crevicular fluid. Not all measures reversed following clinical resolution of the localised inflammation. In particular, chemoattractant levels in GCF appeared to remain elevated at day 35, as did the functional chemotactic responses of peripheral blood neutrophils from healthy volunteers to GCF collected from test sites, which may have implications for gingivitis as a predisposing factor for periodontitis in susceptible individuals.

## Figures and Tables

**Table 1:** Gingival and plaque indices, and GCF volume collected in experimental gingivitis. Percentage of sites above healthy threshold were measured in all volunteers (*n* = 15). GI ranges 0–3 (Loe et al., 1967 [18]); 0 was deemed as absence of gingival inflammation (healthy). The modified Quigley–Hein plaque index ranges 0 to 5; 0 and 1 were deemed to be compatible with health. GCF was collected at non-inflamed (control) and inflamed (test) sites in gingivitis study volunteers. Data are presented as mean ± standard deviation. Statistical test between control and test sites on respective days: paired *t*-test test. * *p* < 0.05, **** *p* < 0.0001. Data are expressed as percentage of data above: 0 for gingival index and 1 for plaque index.

		Day 0	Day 21	Day 35
Gingival Index (% above threshold)	Control	17.78 ± 13.80	21.48 ± 23.93	14.07 ± 10.68
	Test	23.71 ± 15.64	65.19 ± 17.75 ****	17.04 ± 10.17
Plaque Index (% above threshold)	Control	34.22 ± 25.93	21.331 ± 4.31	13.78 ± 11.12
	Test	33.33 ± 19.35	88.89 ± 13.51 ****	16.00 ± 9.71
GCF volume (µL)	Control	0.26 ± 0.15	0.34 ± 0.18	0.20 ± 0.19
	Test	0.31 ± 0.13	0.48 ± 0.14 *	0.19 ± 0.20

**Table 2:** Cytokine quantification from GCF samples. Collected and processed GCF samples from control and test sites for days 0, 21 and 35 were measured for levels of pro-inflammatory host mediators. GCF volumes were normalised according to the amount of protein quantified and data are expressed as amount (pg) per 30 s sample time as previously described (Chapple et al., 1996 [17]). Data are presented as mean ± standard deviation. Statistical test: one-way ANOVA and Tukey’s post-test. * *p* < 0.05, ** *p* < 0.01, *** *p* < 0.0001.

Analyte		Day 0	Day 21	Day 35
C5C5a	Control	6.41 ± 9.29	6.68 ± 3.68	6.81 ± 7.91
	Test	4.62 ± 2.59	7.42 ± 4.82	9.55 ± 6.82 *
CXCL1/GRO-α	Control	0.17 ± 0.18	0.16 ± 0.06	0.16 ± 0.09
	Test	0.29 ± 0.39	0.24 ± 0.14	0.46 ± 0.24 *
CXCL5/ENA-78	Control	0.011 ± 0.009	0.015 ± 0.007	0.013 ± 0.009
	Test	0.018 ± 0.021	0.02 ± 0.006	0.041 ± 0.022 *
IL-1β	Control	0.03 ± 0.04	0.02 ± 0.02	0.04 ± 0.03
	Test	0.04 ± 0.05	0.28 ± 0.2 ***	0.11 ± 0.09
S100A8	Control	1.02 ± 1.68	1.15 ± 0.66	0.43 ± 0.36
	Test	0.82 ± 0.6	1.02 ± 0.47	1.07 ± 0.86
TNF-α	Control	0.002 ± 0.002	0.002 ± 0.001	0.003 ± 0.002
	Test	0.003 ± 0.002	0.004 ± 0.002	0.007 ± 0.004 **
C3a	Control	3.67 ± 3.67	3.49 ± 2.04	1.66 ± 1.37
	Test	3.57 ± 2.92	3.15 ± 3.06	5.21 ± 1.97 *
CXCL2/GRO-β	Control	0.029 ± 0.032	0.031 ± 0.018	0.031 ± 0.028
	Test	0.049 ± 0.043	0.059 ± 0.051	0.08 ± 0.049
CXCL8/IL-8	Control	0.13 ± 0.2	0.08 ± 0.03	0.11 ± 0.09
	Test	0.21 ± 0.32	0.11 ± 0.05	0.4 ± 0.35 **
S100A9	Control	3.65 ± 3.81	3.88 ± 2.29	4.68 ± 3.91
	Test	6.06 ± 5.36	12.71 ± 7.46 **	9.68 ± 6.24
MPO	Control	7.72 ± 8.64	7.71 ± 6.87	9.04 ± 6.49
	Test	12.72 ± 14.56	21.92 ± 14.75	30.74 ± 25.98
S100A9/S100A8	Control	7.08 ± 5.94	4.28 ± 2.84	9.92 ± 6.05
	Test	9.86 ± 7.88	12.04 ± 3.85 *	12.09 ± 7.49

## Data Availability

The datasets used and analysed in this study are available from the corresponding author upon request.

## Supplementary Materials

The following supporting information can be downloaded at: https://www.mdpi.com/article/10.3390/ijerph19106339/s1, Figure S1: Cytokine quantification from GCF samples.
