# Supplementary material for: Impact of Gingivitis on Circulating Neutrophil Reactivity and Gingival Crevicular Fluid Inflammatory Proteins
Source: Int J Environ Res Public Health. 2022 May 23;19(10):6339. doi: 10.3390/ijerph19106339 (PMC9141451; doi:10.3390/ijerph19106339)
Supplement: Supplementary file 1 [file ijerph-19-06339-s001.zip › ijerph-1690152-supplementary.pdf]

International Journal of
*Environmental Research and Public Health*

MDPI

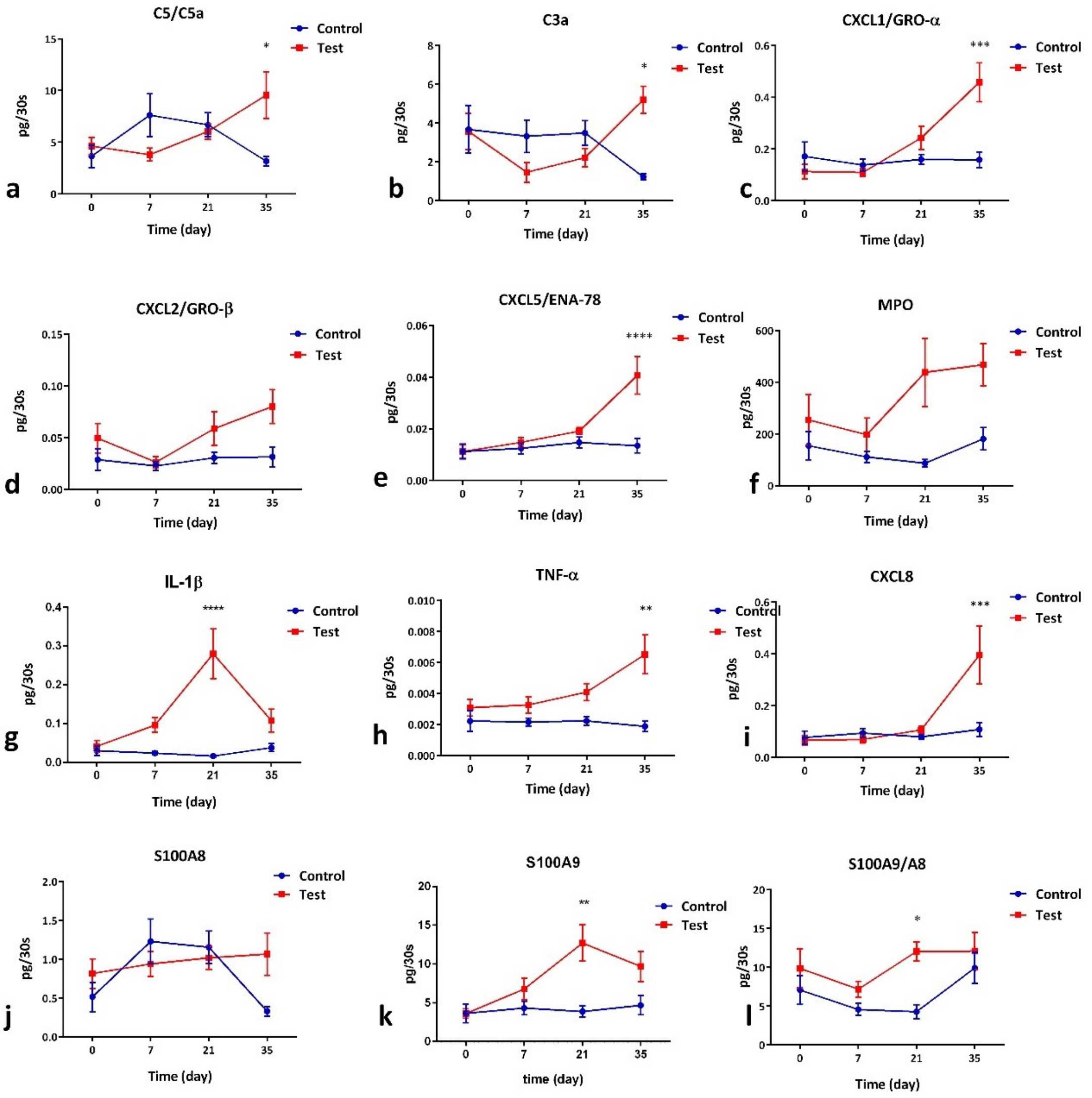


**Supplementary Figure S1.** Cytokine quantification from GCF samples. a. C5/5a; b. C3a, c. CXCL1/GROalpha; d. CXC2/GRObeta; e. CXCL5, ENA-78; f. MPO (myeloperoxidase); g. IL-1beta; h. TNF-alpha; i. CXCL8; j. S100A8; k. S100A9; l. S100A9/S100A8. Collected and processed GCF samples from control and test sites for days 0, 21 and 35 were measured for levels of pro-inflammatory host mediators. GCF volumes were normalised according to the amount of protein quantified and data is expressed as amount (pg) per 30 second sample time as previously described (Chapple et al. 1996). Data presented as mean, +/- standard deviation. Statistical test: One Way ANOVA and Tukeys post-test. * = ($p < 0.05$), ** = ($p < 0.01$), ***= ($p < 0.001$), **** = ($p < 0.0001$).
